# Supplementary figures and images for: High-Frequency Hearing Is Required to Compute a Topographic Map of Auditory Space in the Mouse Superior Colliculus
Source: eNeuro. 2022 May 13;9(3):ENEURO.0513-21.2022. doi: 10.1523/ENEURO.0513-21.2022 (PMC9116932; doi:10.1523/ENEURO.0513-21.2022)

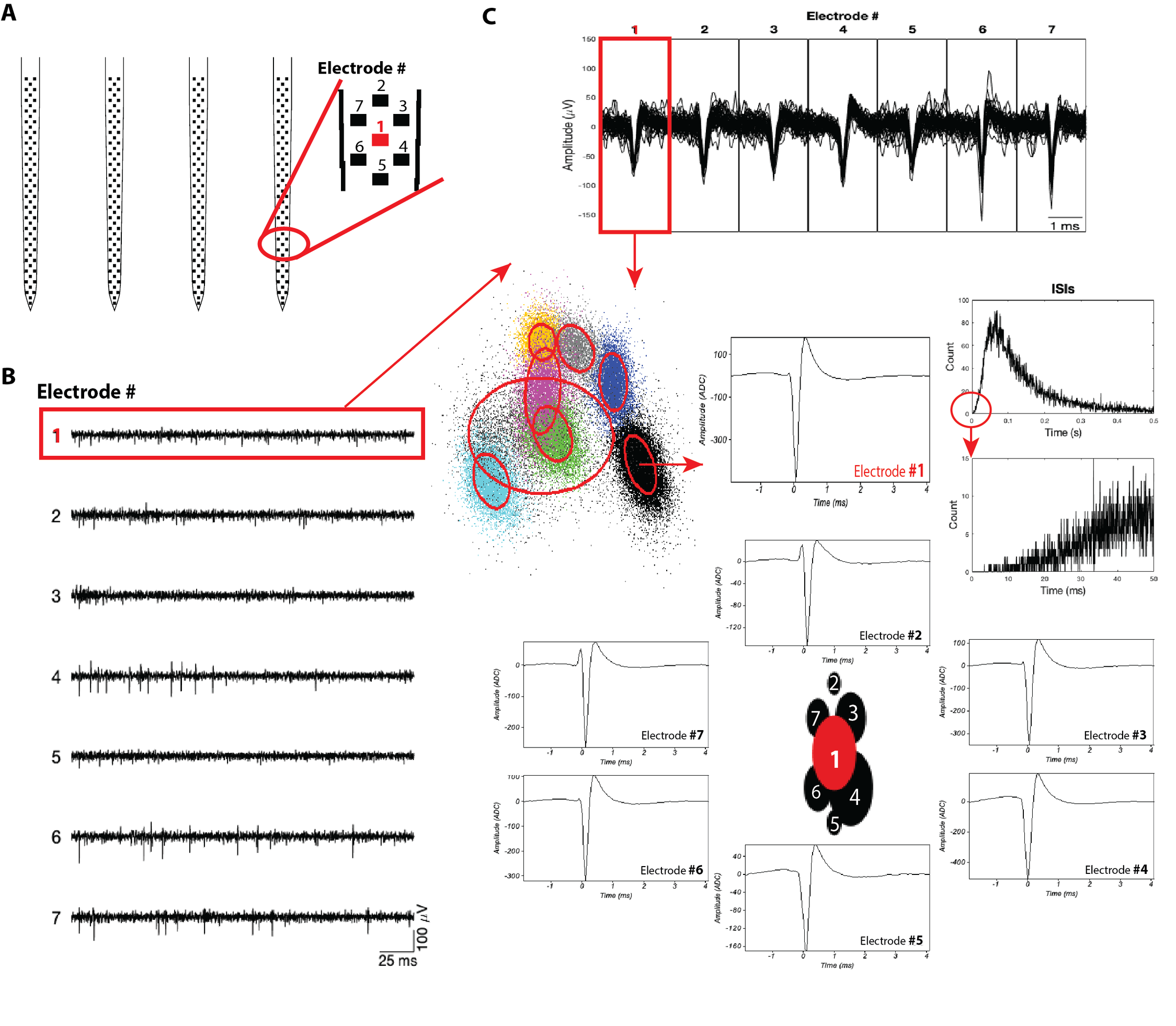

Supplement: Extended Data Figure 1-1 — Silicon probe schematics, examples of raw electrophysiology data, and demonstration of the spike-sorting process. A, A schematic of the 256-channel silicon probes. An example seed electrode is labeled as electrode #1 (red), and its nearest neighbor electrodes are labeled as electrodes #2–7. B, High-pass filtered raw data traces from the example electrodes. C, Spike-sorting process that results in single unit activities. Top, Superimposed 50 example traces of spike waveforms produced on the seed electrode and its nearest neighbors. Middle, Left, scatter plot of the two most significant variables (the data have five dimensions and only two are shown) as found by PCA for the spike waveform data. Middle, The averaged waveform of the black cluster (see the red arrow) recorded on the seed electrode. Right, Interspike-interval histograms (top, 500-ms time scale; bottom, 50-ms time scale) demonstrate no contaminated spikes in this cluster (there is no spike within the ∼1.5-ms refractory period for all detected spikes in this cluster). Bottom, The electrode image of this example cluster [center, the major axis lengths of the ellipses are proportional to the maximum (negative) signal amplitudes] and waveforms of the spikes that belong to the same cluster but detected on the surrounding electrodes. In this specific example, the cyan, dark gray, green and magenta clusters are removed due to contamination (refractory period violation), and the black, blue, gray, and yellow clusters are manually merged and used in subsequent analyses. Download Figure 1-1, TIF file [file enu-eN-NWR-0513-21-s02.tif]

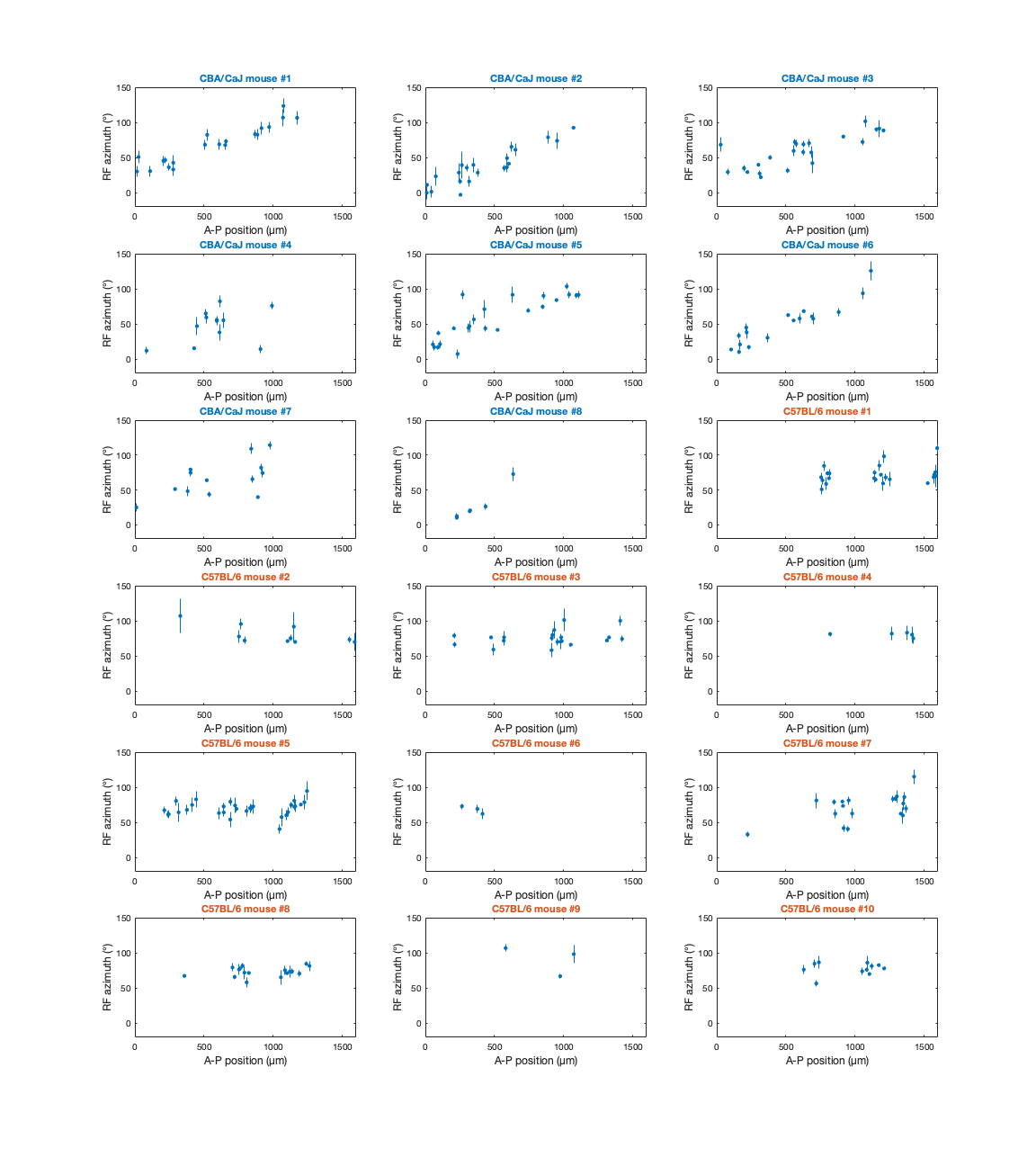

Supplement: Extended Data Figure 1-2 — Topographic maps of auditory space in dSC from individual mice of the CBA/CaJ and C57BL/6 strain. Blue, CBA/CaJ mice; red, C57BL/6 mice. Download Figure 1-2, TIF file [file enu-eN-NWR-0513-21-s03.tif]

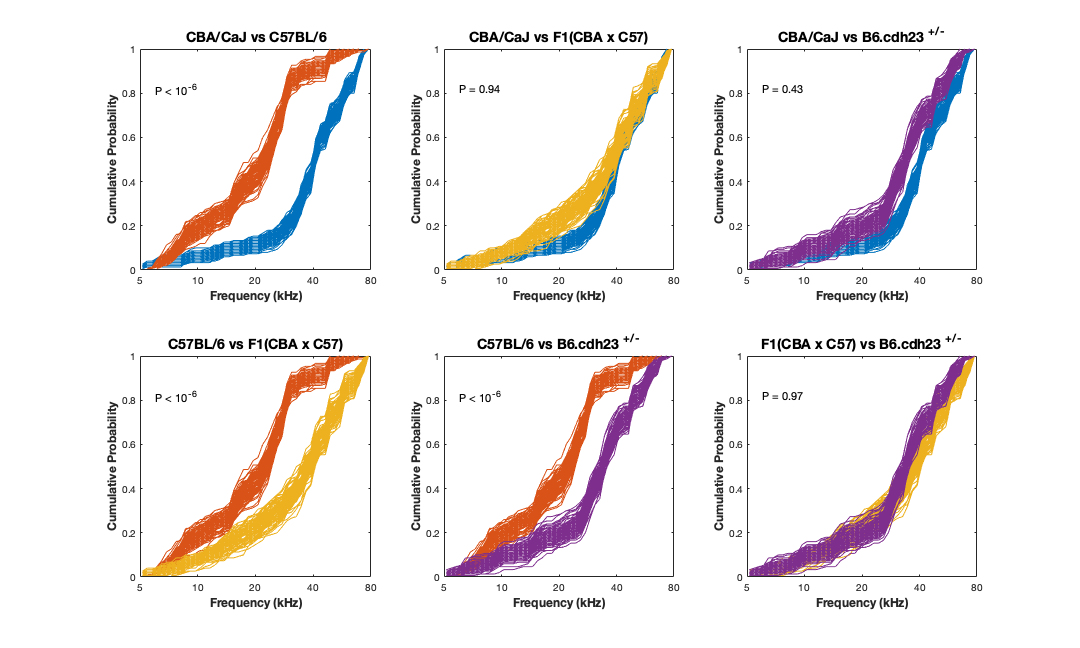

Supplement: Extended Data Figure 2-1 — Results from bootstrapping Kolmogorov–Smirnov test for the cumulative frequency tuning curves. Each panel shows the comparison between a pair of strains. In each panel, 100 trials of the resampled data from the original data of each strain are plotted. Red, C57BL/6; blue, CBA/CaJ; yellow, F1(CBA/CaJ x C57BL/6); purple, B6.cdh23+/–. Download Figure 2-1, TIF file. [file enu-eN-NWR-0513-21-s04.tif]

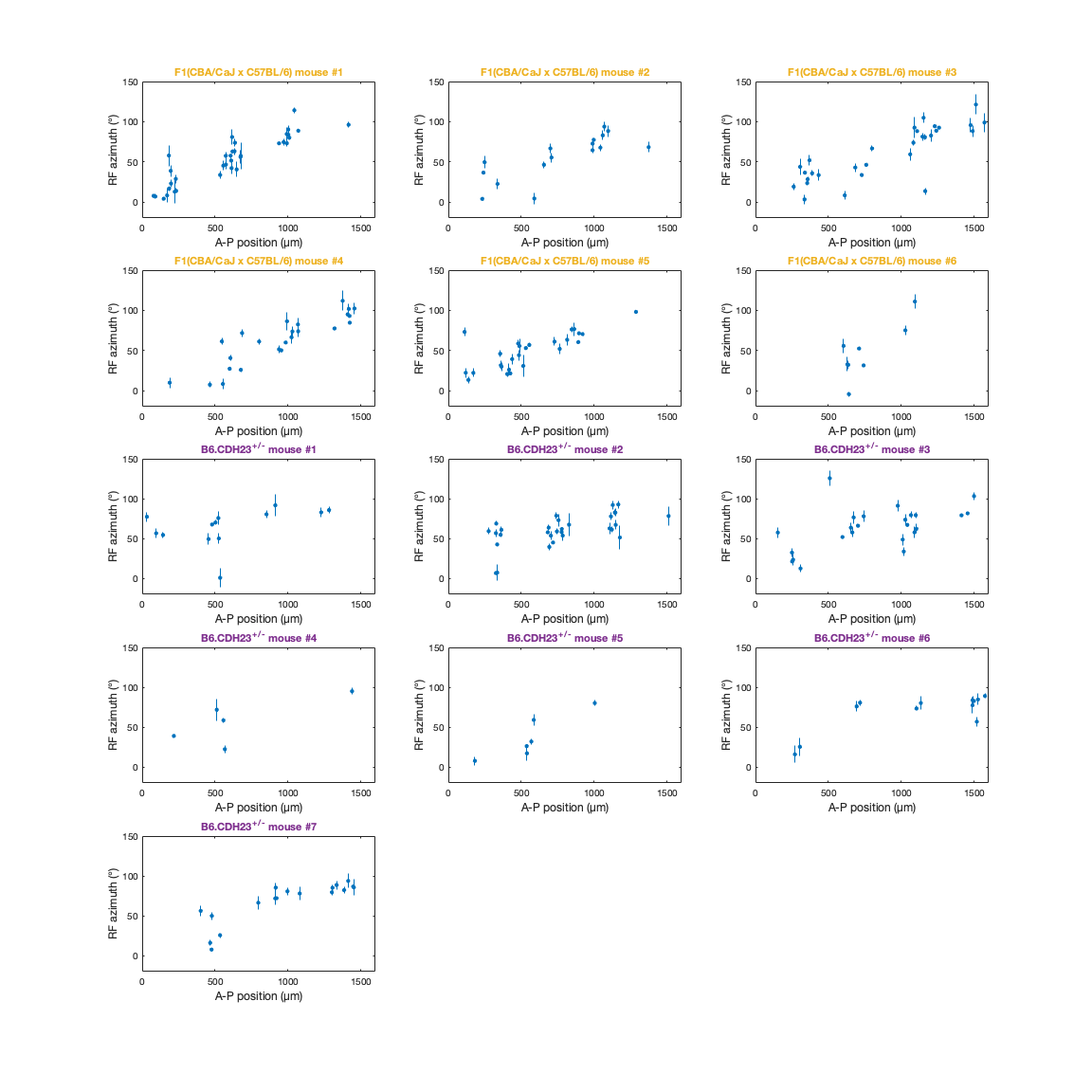

Supplement: Extended Data Figure 4-1 — Topographic maps of auditory space in dSC from individual mice of the F1(CBA/CaJ x C57BL/6) and B6.cdh23+/– strain. Blue, F1(CBA/CaJ x C57BL/6) mice; red,B6.cdh23+/– mice. Download Figure 4-1, TIF file. [file enu-eN-NWR-0513-21-s05.tif]
